# Two Regions with Different Expression of Lipogenic Enzymes in Rats’ Posterior Subcutaneous Fat Depot

**DOI:** 10.3390/ijms252111546

**Published:** 2024-10-27

**Authors:** Jacek Turyn, Ewa Stelmanska, Sylwia Szrok-Jurga

**Affiliations:** Department of Biochemistry, Faculty of Medicine, Medical University of Gdansk, 80-211 Gdansk, Poland; ewa.stelmanska@gumed.edu.pl

**Keywords:** subcutaneous adipose tissue, brown adipose tissue, fatty acid synthase, ATP-citrate lyase, malic enzyme, lipogenic enzymes, mRNA expression, uncoupling protein 1

## Abstract

Lipid metabolism in various adipose tissue depots can differ vastly. This also applies to lipogenesis, the process of synthesizing fatty acids from acetyl-CoA. This study compared the expression of some lipogenic enzymes: fatty acid synthase (FASN), ATP-citrate lyase (ACLY), and malic enzyme 1 (ME1) in different regions of the posterior subcutaneous adipose tissue in rats. Methods and Results: Posterior subcutaneous adipose tissue collected from twelve-month-old Wistar rats was divided into six parts (A–F). The expression of genes encoding lipogenic enzymes was assessed by measuring their activity and mRNA levels using real-time PCR. In the gluteal region of the fat pad, there were much higher levels of activity and mRNA for these lipogenic enzymes compared to the dorsolumbar region. The mRNA level of FASN increased by more than twentyfold, whereas the level of ME1 and ACLY increased eight- and fivefold respectively. This phenomenon was observed in both old and young animals. Furthermore, the lack of uncoupling protein one (Ucp1) expression suggests that neither the presence of brown adipocytes in the gluteal part nor the transformation of white adipocytes into beige contributed to the observed differences. Conclusion: These results indicate that the gluteal white adipose tissue appears to be a unique and separate subcutaneous fat depot.

## 1. Introduction

**Figure 1:**
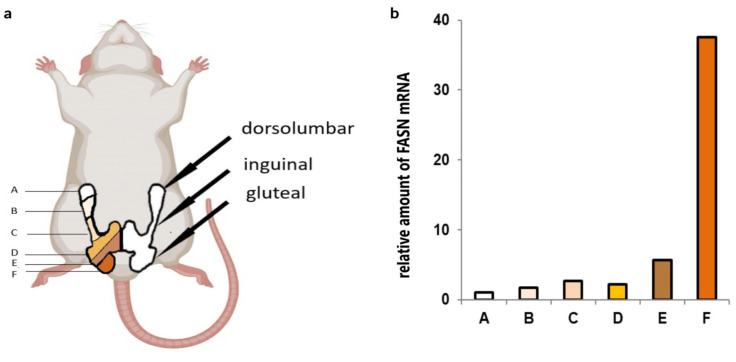
Outline of localization of rat posterior subcutaneous white adipose tissue (WAT). The schematic diagram depicts the location of three distinct regions in this pad of adipose tissue: dorsolumbar (WATd), inguinal (WATi), and gluteal (WATg) fragments obtained as a result of cutting marked with letters A to F (**a**). Preliminary assessment of the amount of fatty acid synthase (FASN) mRNA in a twelve-month-old rat’s posterior subcutaneous fat pad is divided into six parts: A is the part closest to the head, and F is closest to the tail. Data are presented as ratio vs. value in part A (**b**). This figure was prepared partially with the Bio-render usage.

Understanding how adipose tissue functions is essential for obesity treatment and related metabolic diseases. Differences in the metabolism of individual fat pads in animal models are fundamental to the correct interpretation of experimental results [1]. We can distinguish three types of adipose tissue in mammals. White adipose tissue (WAT) plays a crucial role in maintaining the body’s energy balance by acting as the primary site for the storage of triglycerides and the release of fatty acids [2]. Brown adipose tissue (BAT) and beige adipose tissue (BeAT) are characterized by abundant mitochondria. The presence of the distinctive uncoupling protein 1 (Ucp1) significantly contributes to their role in thermogenesis [2,3,4,5]. Some authors also distinguish pink adipose tissue that appears only during pregnancy and lactation in the mammary gland [6,7]. Adipose tissue is anatomically divided into two main subcutaneous and visceral parts. In rodents, each part comprises several depots. The subcutaneous adipose tissue can be further categorized into the anterior pad, located in the front part of the body around the forelimbs and neck, and the posterior pad, situated in the area of the lower limbs. In the part located on the posterior side of the body, there are three distinct areas with unclear boundaries: the dorsolumbar (WATd), the inguinal (WATi), and the gluteal region (WATg) (Figure 1a) [6,7,8,9,10]. The anterior subcutaneous fat comprises both WAT and BAT components. The interscapular BAT depot is the primary site of BAT distribution. In contrast, the posterior subcutaneous fat pad is identified as WAT [6,7,11]. However, other types of adipocytes may appear within the depots, either as a result of the de novo differentiation of precursor cells or the transdifferentiation of white adipocytes into beige (beigeing) [12,13], or, as other researchers claim, as a result of the reversible conversion of white to brown adipocytes (browning) [7,14]. Thus, the quantity of various adipocyte types in adipose tissue fluctuates and is influenced by factors such as physical activity, cold exposure, β3-adrenergic agonists, thyroid hormones, and peroxisome proliferator-activated receptor gamma (PPARγ) activators [6,7,12,13,14,15]. Additionally, WAT adipocytes are not a homogenous group. Those located in visceral fat depots differ from subcutaneous adipocytes regarding gene expression, adipokine secretion intensity, and their response to excessive food supply [11].

Acetyl-CoA metabolism is an essential component in lipid homeostasis [16,17]. Lipogenesis involves the synthesis of fatty acids from acetyl-CoA and is primarily observed in the liver and adipose tissue. However, the enzymes responsible for this process are present in numerous other organs within the body [18]. In rats, the expression of mRNA and the activity of lipogenic enzymes can vary significantly depending on the type and location of adipose tissue [19,20,21,22,23]. For instance, in BAT, the activities of fatty acid synthase (FASN), ATP-citrate lyase (ACLY), and cytosolic malic enzyme 1 (ME1) are higher than in WAT [19]. Furthermore, the expression of genes that encode lipogenic enzymes in different fat pads shows differential responses to factors such as diet, hormones, and age. Kochan et al. demonstrated that multiple alternating starvation–refeeding cycles in WAT increase lipogenic enzyme activity. In contrast, this effect does not occur in BAT [1]. Rats subjected to thirty days of food restriction exhibit an increased expression of lipogenic enzymes in WAT. This effect is notably heightened when, following a period of restricted feed access, the animals are allowed to feed ad libitum for two days [18,21,24]. Previous publications indicate that in posterior subcutaneous WAT, the impact of food restriction on the expression of two lipogenic enzymes, stearoyl-CoA desaturase 1 and elongase 6, is significantly greater than in retroperitoneal WAT (the depot of visceral WAT) [25]. The age of the animals significantly influences the response of WAT to long-term caloric restriction and fasting/refeeding [21,22]. The measurement of FASN activity in randomly collected subcutaneous WAT fragments from aged rats yielded variable results. Within a homogeneous group of animals, the differences between samples were noteworthy, with the highest value exceeding the lowest by more than tenfold. This observation prompted an investigation into the uniformity of gene expression encoding lipogenic enzymes in rat posterior subcutaneous adipose tissue.

## 2. Results

The expression of the *Fasn* gene in the posterior subcutaneous WAT of twelve-month-old rats exhibited significant heterogeneity. The fat pad obtained from one side of the body (*n* = 3) was partitioned into six equal segments labeled as A–F, where A represents the most anterior region and F represents the most posterior part. Subsequently, there were no discernible differences in FASN mRNA levels between segments A–D. However, in the following segment, E, the expression was nearly sixfold higher. Finally, in segment F, the expression of this gene was approximately almost fortyfold higher compared to segment A (Figure 1b). The segments demonstrating elevated mRNA levels of the gene encoding FASN roughly correspond to the region identified as WATg (Figure 1a).

**Figure 2:**
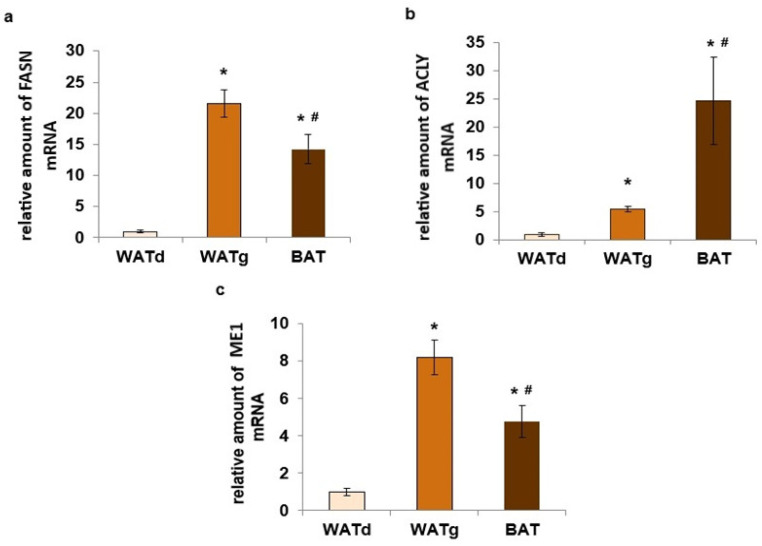
Increased expression levels of lipogenic genes *Fasn* (**a**), *Acly* (**b**), and *Me1* (**c**) in the gluteal region (WATg) of posterior subcutaneous white adipose tissue (WAT) and interscapular brown adipose tissue (BAT) of twelve-month-old rats. The data are presented as mean fold change relative to dorsolumbar part (WATd) of posterior subcutaneous WAT ± SEM; * *p* < 0.05 vs. dorsolumbar part of posterior subcutaneous WAT; # *p* < 0.05 vs. gluteal part of posterior subcutaneous WAT.

**Figure 3:**
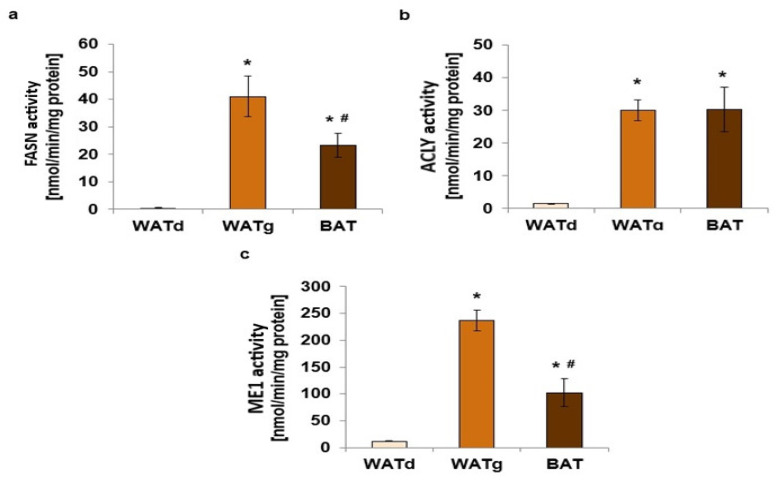
Increased activity of lipogenic enzymes FASN (**a**), ACLY (**b**), and ME1 (**c**) in gluteal white adipose tissue (WATg) and interscapular brown adipose tissue (BAT) of twelve-month-old rats. The data are presented as mean ± SEM; * *p* < 0.05 vs. dorsolumbar part of posterior subcutaneous (WATd); # *p* < 0.05 vs. WATg.

An examination of a larger group of rats confirmed this observation. The FASN mRNA levels in WATg were consistently more than twenty times greater than those in WATd, as demonstrated in Figure 2a. Moreover, the amount of mRNA of two other genes encoding lipogenic enzymes, ACLY and ME1, in the gluteal subcutaneous part of WAT also differed significantly (Figure 2b,c). Similar differences were observed when comparing WATd and another depot of subcutaneous fat-interscapular BAT. In addition, the measurements of the activity of these three lipogenic enzymes confirm their much higher expression in WATg and BAT compared to WATd (Figure 3a–c). The analyses indicated that the WATg expression and activity levels of the FASN and ME1 were even higher than in BAT (Figure 2a,c and Figure 3a,c). Although the quantity of ACLY mRNA was greater in BAT (Figure 2b), there was no significant difference in enzyme activity between WATg and BAT (Figure 3b).

**Figure 4:**
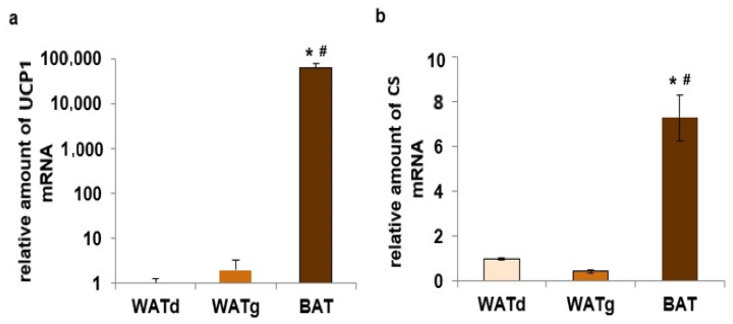
Expression levels of *Ucp1* (**a**) and *Cs* (**b**) genes in dorsolumbar white adipose tissue (WATd), gluteal white adipose tissue (WATg), and interscapular brown adipose tissue (BAT) of twelve-month-old rats. The data are presented as mean fold change relative to the dorsolumbar part of posterior subcutaneous WAT ± SEM; * *p* < 0.05 vs. WATd; # *p* < 0.05 vs. WATg.

**Figure 5:**
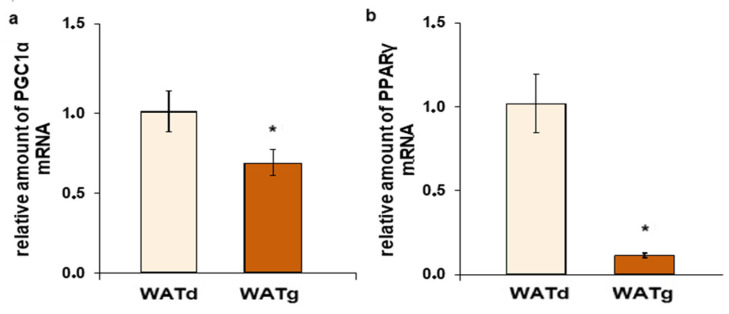
Expression levels of *Pgc1*α (**a**) and *Pparγ* (**b**) genes in dorsolumbar white adipose tissue (WATd) and gluteal white adipose tissue WATg of old (twelve-month-old) rats. The data are presented as mean fold change relative to the dorsolumbar part of posterior subcutaneous WAT ± SEM; * *p* < 0.05 vs. WATd.

The *Ucp1* gene was strongly expressed in BAT adipocytes of twelve-month-old rats. In contrast, only trace amounts of this gene mRNA were observed in both subcutaneous WAT fragments (Figure 4a). The quantity of mitochondrial marker number citrate synthase (CS) mRNA increased seven-fold in BAT compared to WATd and WATg, while *Cs* gene expression between these two WATs fragments did not differ significantly (Figure 4b). In addition, we observed statistically significant reduced levels of peroxisome proliferator-activated receptor gamma coactivator 1-alpha (PGC1-α) and PPARγ mRNA in WATg compared to WATd (Figure 5a,b).

**Figure 6:**
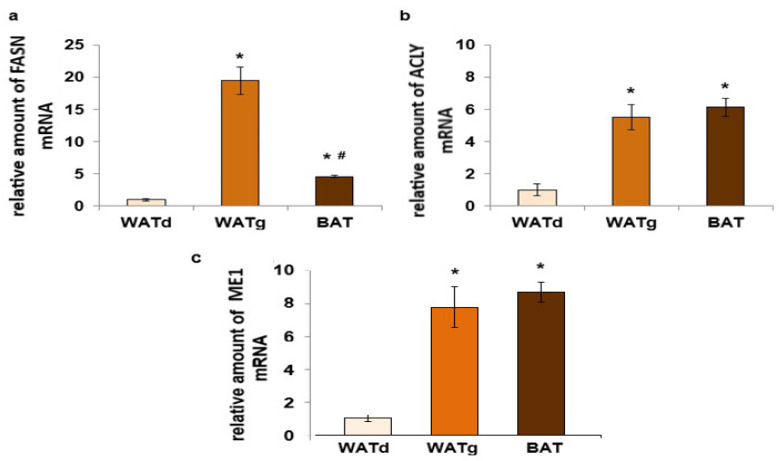
Expression levels of *Fasn* (**a**), *Acly* (**b**), and *Me1* (**c**) genes in dorsolumbar white adipose tissue (WATd), gluteal white adipose tissue (WATg), and interscapular brown adipose tissue (BAT) of young (two-month-old) rats. The data are presented as mean fold change relative to the dorsolumbar part of posterior subcutaneous WAT ± SEM; * *p* < 0.05 vs. WATd; # *p* < 0.05 vs. WATg.

**Figure 7:**
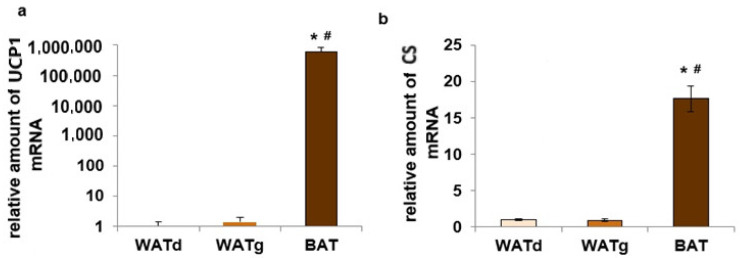
Expression levels of *Ucp1* (**a**) and *Cs* (**b**) genes in dorsolumbar white adipose tissue (WATd), gluteal white adipose tissue (WATg), and interscapular brown adipose tissue (BAT) of two-month-old rats. The data are presented as mean fold change relative to the dorsolumbar part of posterior subcutaneous WAT ± SEM; * *p* < 0.05 vs. WATd; # *p* < 0.05 vs. WATg.

**Figure 8:**
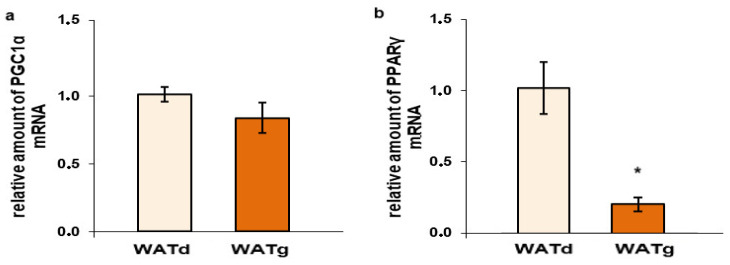
Expression levels of *PGC1α* (**a**) and *PPARγ* (**b**) gene in dorsolumbar white adipose tissue (WATd) and gluteal white adipose tissue (WATg) of young (two-month-old) rats. The data are presented as mean fold change relative to the dorsolumbar part of posterior subcutaneous WAT ± SEM; * *p* < 0.05 vs. WATd.

Measurements performed on tissues obtained from two-month-old rats indicated that the expression of the selected lipogenic enzymes FASN, ACLY, and ME1 significantly differed between WATd and the second, marginal fragment of posterior subcutaneous fat-WATg and, as in year-old rats, was considerably higher in WATg (Figure 6). As expected, the mRNA levels of these genes were higher in BAT than those observed in WATd. However, *Fasn* gene expression was lower in BAT compared to WATg (Figure 6a), while the amount of ACLY and ME1 mRNA in these tissues was comparable (Figure 6b,c). The mRNA levels of the *Ucp1* gene in WATd and WATg were near the detection limit, similar to those of old rats, in contrast to BAT (Figure 7a). Notably, a comparable, low level of *Cs* gene expression was evident in both analyzed segments of subcutaneous WAT, while BAT exhibited an mRNA level of this gene more than 15 times higher compared to WATd and WATg (Figure 7b). Regarding PGC1α, we did not detect statistically significant differences in expression levels between WATg and WATd (Figure 8a). However, similar to old rats, we observed a decreased mRNA level of the gene encoding PPARγ in WATg in comparison to WATd (Figure 8b).

## 3. Discussion

The results above demonstrate the metabolic heterogeneity of posterior subcutaneous adipose tissue in male rats. The expression levels of mRNA and the activity of lipogenic enzymes such as FASN, ACLY, and ME1 are notably higher in the region located at the back of this fat pad compared to other parts, roughly corresponding to the gluteal area. The division of the posterior subcutaneous WAT into three regions (dorsolumbar, inguinal, and gluteal) is only approximate, lacking strict boundaries [6,7,8,9,10]. These findings indicate the need for the verification and detailed characterization of this fat pad. 

Although the entire posterior subcutaneous fat depot is classified as WAT [6,7,9,10,11], the expression level of lipogenic enzymes measured in WATg is similar to those observed in these animals in BAT. Many factors lead to the appearance of brown/beige adipocytes within WAT. In mice, in posterior subcutaneous adipose tissue, brown adipocytes appear as a result of the administration of PPARγ agonists [26]. The exposure of animals to low temperatures over 10 days yields similar effects; however, these effects vary among different strains [27]. Reports indicate the presence of BeAT in certain strains of mice in the WATi of animals with no prior cold exposure [5]. In the fragments of subcutaneous adipose tissue examined herein, no significant differences were observed in the expression of the *Ucp1* gene—characteristic of BAT and BeAT. Furthermore, the mRNA level of this gene in WATg and WATd approaches the detection limit (Figure 4a and Figure 7a).

In adult humans, BAT is primarily located in the neck and supraclavicular regions. In addition to these two locations, it is also found in the paravertebral region, within the mediastinum, particularly in the para-aortic area, surrounding the heart at the apex, and in infra-diaphragmatic depots, notably in the perirenal space [28]. The characterization of BAT from adult humans reveals a molecular profile more comparable to BeAT than to classical BAT [29]. Enhancing BAT-BeAT activity through cold exposure, dietary modifications, or pharmacological interventions is positively associated with energy expenditure. Men exposed to low temperatures for 10 days exhibited improved glucose absorption in BAT, increased glucose oxidation, and enhanced insulin sensitivity [30]. Thiazolidinediones—PPARγ agonists, commonly used as insulin sensitizers for type 2 diabetes—have also been studied for their ability to promote thermogenic gene expression in both white and brown adipocytes. However, their application has been limited by the presence of undesirable side effects [29]. It was revealed that, unlike in mice, in humans, exercise does not induce the browning of subcutaneous adipose tissue [31]. Interestingly, the browning of WATs in humans has been observed in response to severe burn injury [32]. As PPARγ and PGC1α are extensively expressed in adipose tissues, and their increased expression is linked with browning/beigeing and adipogenesis [33,34], we determined the mRNA level of these genes in WATd and WATg in old and young rats. According to the obtained results, we can assume that differences in lipogenic enzyme expression in marginal sides of WATs are not the result of browning, beigeing, or adipogenesis processes.

Moreover, our examination included the determination of the expression of CS, a Krebs cycle enzyme. The results revealed no differences in CS mRNA level between the analyzed regions of the subcutaneous WAT pad, indicating no significant differences in the number of mitochondria. However, it was observed that the quantity of CS mRNA was notably decreased compared to BAT (Figure 4b and Figure 7b). Consequently, the increased expression of lipogenic genes in WATg cannot be attributed to the presence of BAT/BeAT adipocytes.

Recent studies suggest that WAT adipocytes are heterogeneous rather than a homogeneous group of cells. In a study by Lee KY et al. [35] conducted in mice, three types of white adipocytes derived from separate preadipocyte lines were distinguished. Each of these types was found to have different metabolic and regulatory properties. In vitro experiments revealed that these types differ in the de novo synthesis of fatty acids in response to insulin. It is important to note that individual fat deposits vary significantly in the content of particular types of adipocytes. In all visceral fat pads, all three types of adipocytes can be found in different proportions, whereas in subcutaneous WAT, only two types were found. However, in each cluster, there is a population of adipocytes that is not characterized by the authors, which increases the complexity of this tissue [35]. Thus, the properties of individual fat deposits would result from the participation of particular types of adipocytes in them.

Age significantly affects the functionality of various organs and tissues, including adipose tissue, where it is associated with changes in adipocyte size and overall weight, as well as alterations in lipid composition [36,37]. As the aging process progresses, the expression of genes related to lipogenesis also changes. The retroperitoneal depot of visceral WAT in 3.5-month-old rats exhibited increased activity of FASN and ME1 in comparison to 12-month-old rats, as demonstrated by the research conducted by Mooradian and Albert [38]. An analogous phenomenon in this fat pad was observed when the level of expression of other lipogenic enzymes was compared (ACL, acetyl-CoA carboxylase, glucose-6-phosphate dehydrogenase, and 6-phosphogluconate dehydrogenase) in 2- and 20-month-old rats [39]. The expression of *Fasn* and *Me1* genes significantly decreased between 2 and 6 months of age in retroperitoneal WAT, as well as in epididymal WAT (another visceral fat pad) and posterior subcutaneous WAT [40]. Based on the analysis of the relative mRNA levels of lipogenic enzymes in old and young rats, we compared how aging affects the expression of FASN, ME1, and ACL. Consistent with prior research, the mRNA levels of lipogenic enzymes decreased in 12-month-old compared to 2-month-old rats (Figure S1). However, despite the alteration in lipogenic gene expression with age, the studies of WATd and WATg presented herein indicate that the proportions of these variations remain constant. The differences in FASN mRNA expression between WATg and WATd in 12-month-old rats were nearly indistinguishable from that observed in 2-month-old rats. Moreover, the differences in Acly and Me1 mRNA levels between the two marginal WATs regions were also comparable in both aged and juvenile animals.

An examination of *Ucp1* and *Cs* genes expression reveals that the variations between WATg and WATd in both 12- and 2-month-old animals are not associated with WAT browning (or beigeing). Nevertheless, as adipocytes age, they increasingly adopt a phenotype resembling white adipocytes, therefore inhibiting the process of adipocyte browning in older individuals [41].

Older animals were significantly heavier and had statistically more adipose tissue in comparison to younger rats. The mass of the posterior WATs was more than fourfold greater in old than in young animals (Table 1). According to the above-mentioned results, we may speculate that WATd and WATg exhibit analogous changes with age, and the process of lipogenesis is reduced to a comparable extent.

Defining the boundaries of fat pads in posterior subcutaneous adipose tissue can be challenging. Bagchi et al.’s meticulous publication provides an exceptional description that facilitates the precise delineation of these boundaries and enables appropriate tissue preparation [42]. Our results suggest that the subcutaneous adipose tissue’s gluteal part in male rats should be considered as a distinct WAT depot with different lipid metabolism. Additionally, under thermal comfort conditions, there is no evidence of the presence of brown/beige adipocytes. Although the adipocytes in the posterior fat pads of rodents and humans differ in size and metabolic activity [43], an understanding of the functional heterogeneity of the inguinal fat in rodents may offer insights into the metabolic adaptations and potential risks associated with obesity and related disorders.

## 4. Materials and Methods

### 4.1. Animals and Tissue Collection

Twelve-month-old (*n* = 11) and two-month-old (*n* = 7) male Wistar rats were maintained at 22 °C under a light/dark (12:12 h) cycle with lights on at 7:00 a.m. and fed with a commercial diet with unlimited access to food and tap water [24]. The animals were anesthetized and killed by decapitation (between 8:00 and 10:00 a.m.). The interscapular BAT and the posterior subcutaneous WAT were excised and immediately frozen in the liquid nitrogen and then stored at −80 °C until analysis. The subcutaneous adipose tissue from one side was divided into three parts before freezing. Two extreme ones, corresponding to dorsolumbar and gluteal portions, were collected. In the case of one twelve-month-old animal, the posterior subcutaneous WAT was divided into six approximately equal parts (Figure 1) that were immediately frozen. The animals’ weights, the WAT mass, and the posterior subcutaneous WAT mass are presented in Table 1.

### 4.2. Enzyme Activity Assay

Approximately 0.2 g of tissue was placed in 1.5 mL ice-cold 20 mmol/LTris hydrochloride buffer (pH 7.8) containing 0.2% Triton X-100. The tissue was homogenized manually with a Teflon pestle homogenizer and centrifuged at 30,000× *g* for 20 min. The resulting supernatant was decanted, and the pellet was resuspended in 0.5 mL of isolation medium, homogenized again, and centrifuged as before. The supernatant was combined with the one obtained after the first centrifugation step and used for the enzyme assay. The activities of FASN (EC 2.3.1.85), ACLY (EC 4.1.3.8), and ME1 (EC 1.1.1.40) were measured as described previously [44]. All assays were duplicated at 37 °C using a Beckman DU 68 spectrophotometer (Beckman Coulter Inc, Brea, CA, USA). The absorbance changes, both against time and against enzyme concentration, were linear. Protein assays were performed according to Peterson’s method [45].

### 4.3. RNA Isolation

Total cellular RNA was extracted from frozen tissue with PureZOL™ RNA Isolation Reagent (Bio-Rad Laboratories Inc, Hercules, CA, USA) according to the manufacturer’s protocol.

### 4.4. cDNA Synthesis

First-strand cDNA was synthesized from 4 μg of total RNA (RevertAid TM First Strand cDNA Synthesis Kit—Thermo Fisher Scientific Inc, Lenexa, KS, USA). Prior to the amplification of cDNA, each RNA sample was treated with RNase-free DNase I (Thermo Fisher Scientific, Inc.) at 37 °C for 30 min.

### 4.5. Real-Time PCR 

FASN, ACLY, ME1, UCP1, CS, PGC1α, and PPARγ mRNA levels were quantified by real-time PCR using CFX96™ Maestro 2.3 Real-Time PCR Detection System (Bio-Rad Laboratories Inc). Primers were designed with Primer-BLAST software (https://www.ncbi.nlm.nih.gov/tools/primer-blast/ accessed on 1 August 2024) [46] from gene sequences obtained from the Nucleotide database (https://www.ncbi.nlm.nih.gov/nucleotide/ accessed on 1 August 2024). The list of oligonucleotide primer pairs used in this work is found in Table 2. Real-time PCR amplification was performed in a 20 μL volume using iQ SYBR Green Supermix (Bio-Rad Laboratories). Each reaction contained cDNA and 0.3 μM of each primer. Samples were incubated for an initial denaturation and polymerase activation at 95 °C for 5 min, followed by 35 PCR cycles of amplification (92 °C for 20 s, 57 °C for 20 s, and 72 °C for 30 s). All the samples were run in triplicate. Control reactions with no template cDNA added were performed for each assay. TATA-box binding protein (TBP) and ribosomal protein L19 (RPL19) mRNA were quantified in corresponding samples to compensate for variations in the amount of added RNA and the reverse transcription efficiency. The results were normalized to these values. Since the results obtained using TBP or RPL19 were very similar, the results section presents only the results as quantified mRNA/Tpb. Relative quantities of transcripts were calculated using the 2^−ΔΔCT^ formula [47]. The results are expressed in arbitrary units, with one unit being the mean mRNA level determined in WATd. The amplification of specific transcripts was further confirmed by obtaining melting curve profiles and subjecting the amplification products to 1% agarose gel electrophoresis.

### 4.6. Statistics

The statistical significance of differences between the groups was verified based on a one-way analysis of variance (ANOVA) with the Tukey post hoc test. Differences between the groups were considered significant at *p* < 0.05. SigmaStat 3.0 software (Sigma Stat Inc., San Jose, CA, USA) was used for all statistical analyses.

## 5. Conclusions

These results indicate that the gluteal WAT appears to be a unique and separate subcutaneous fat depot in rats.

## Figures and Tables

**Table 1:** The body weight, adipose tissue mass (retroperitoneal, epididymal, and subcutaneous), and posterior subcutaneous WAT mass of 2- and 12-month-old rats (mean ± SD).

Group	Body Weight (g)	WAT Mass (g)	Posterior Subcutaneous WAT Mass (g)
2-month-old	270 ± 17.2	4.5 ± 1.2	2.5 ± 0.7
12-month-old	528 ± 56.7 *	30 ± 11.2 **	11.8 ± 6.7 *

WAT—white adipose tissue, * *p* < 0.05 vs. 2-month-old group; ** *p* < 0.01 vs. 2-month-old group.

**Table 2:** Primer sequences.

Gene	Primer Sequence (5′→3′)
*Fasn*	F: ATGGGAAGGTGTCTGTGCACAT R: TGTGGATGATGTTGATGATA
*Acly*	F: CTCACACGGAAGCTCATCAA R: ATGGCAACACCCTCGTAGAC
*Me1*	F: GCCCTGAATATGATGCGTTT R: CACAGACGCTGTTCCTTGAA
*Ucp1*	F: CCGAGCCAAGATGGTGAGTT R: CCTTGGATCTGAAGGCGGAC
*Cs*	F: GTAATTCATCTCCGTCATGCCA R: GTCAGCGAGAGTTTGCTCTGAA
*Tbp*	F: CACCGTGAATCTTGGCTGTAAAC R: ATGATGACTGCAGCAAACCG
*Rpl19*	F: CTGCGTCTGCAGCCATGAGTATGC R: TTACCACAGCGGAGGACGCTAGAG
*PPAR γ*	F: CCAGAGTCTGCTGATCTGCG R: GCCACCTCTTTGCTCTGCTC
*PGC1α*	F: ACTGAGCTACCCTTGGGATG R: GGAATATGGTGATCGGGAAC

## Data Availability

The data obtained are presented in the article, and further inquiries can be directed to the corresponding author: jacek.turyn@gumed.edu.pl.

## Supplementary Materials

The supporting information can be downloaded at: https://www.mdpi.com/article/10.3390/ijms252111546/s1.
