# Supplementary material for: Two Regions with Different Expression of Lipogenic Enzymes in Rats’ Posterior Subcutaneous Fat Depot
Source: Int J Mol Sci. 2024 Oct 27;25(21):11546. doi: 10.3390/ijms252111546 (PMC11546078; doi:10.3390/ijms252111546)
Supplement: Supplementary file 1 [file ijms-25-11546-s001.zip › ijms-3260637-supplementary.pdf]

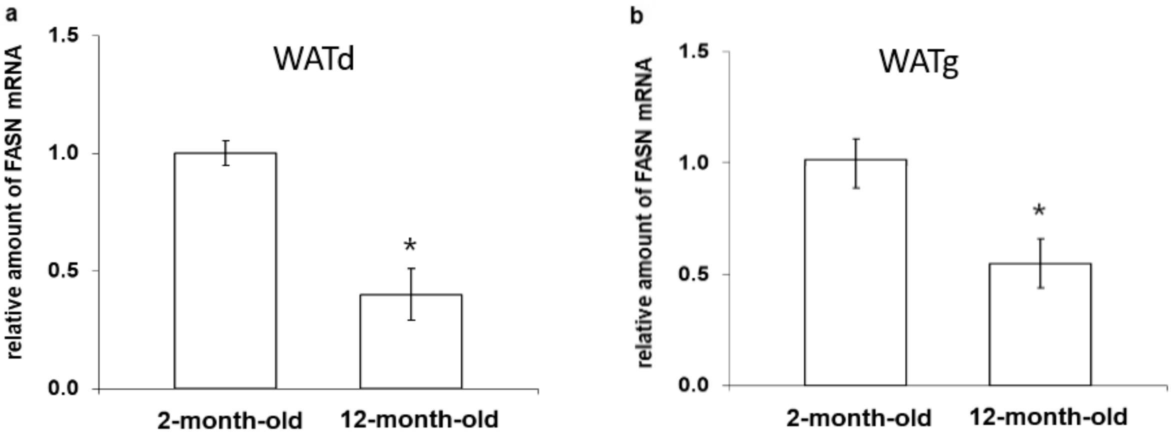

Figure S1 The effect of aging on fatty acid synthase (FASN) mRNA level in posterior subcutaneous white adipose tissue: dorsolumbar (WATd) **(a)** and gluteal (WATg) **(b)**. The data is presented as the mean fold change in aged rats (12 months) compared to the expression in young rats (2 months) ± SEM; * $p < 0.05$.
